# Overdose risk in the context of chemsex among gay, bisexual, and other men who have sex with men

**DOI:** 10.1186/s12954-026-01410-4

**Published:** 2026-02-06

**Authors:** Felipe Duailibe, Mark Hull, Julio Montaner, Aaron Purdie, Viviane D. Lima

**Affiliations:** 1 British Columbia Centre for Excellence in HIV/AIDS, Vancouver, BC Canada; 2 University of British Columbia, Vancouver, BC Canada; 3 Health Initiative for Men (HIM), Vancouver, BC Canada

**Keywords:** Chemsex, Gay, bisexual, and other men who have sex with men, Overdose

## Abstract

Chemsex is defined as the use of psychoactive substances – most commonly methamphetamine, synthetic cathinones, and Gamma-hydroxybutyrate/Gamma butyrolactone (GHB/GBL) – to enhance sexual activity primarily among gay, bisexual, and other men who have sex with men (GBM). It is associated with higher rates of sexually transmitted infections, HIV, and mental health conditions. However, despite substantial public health concerns regarding rising overdose deaths, the relationship between chemsex and overdose remains poorly studied. In this perspective, we synthesize the current evidence, identify critical knowledge gaps regarding the association between chemsex and overdose risk among GBM, and outline harm reduction and behavioral interventions. Assessing chemsex-related overdose deaths among GBM is challenging due to the limited documentation of sexual practices and sexual orientation in medical or legal records. Stigma further reduces disclosure, and chemsex involvement is rarely identifiable posthumously. Chemsex frequency and perceptions of harm also obscure problematic use. Effective pharmacologic treatments for stimulant dependence remain limited. In contrast, contingency management (CM) has been proven effective in reducing stimulant use, yet remains underutilized. Integrated harm reduction approaches are essential to mitigate the potential risks of chemsex. Key interventions include HIV testing, needle exchange, sexual health screenings, psychosocial interventions, and vaccinations. There is an urgent need for targeted research, improved data collection, and tailored harm reduction strategies to better understand and reduce overdose risk within chemsex contexts. Addressing these gaps is essential for reducing preventable deaths and improving health outcomes in this population.

## Introduction

Chemsex, also known as Party and Play (PnP), refers to the use of specific psychoactive substances, primarily among gay, bisexual, and other men who have sex with men (GBM), during sexual activities to enhance, prolong, or facilitate the sexual experience [1–3]. Although there is no consensus on the drugs used in chemsex, potentially due to local drug markets, availability and cost, the literature consistently identifies methamphetamine, Gamma-hydroxybutyrate/Gamma butyrolactone (GHB/GBL), and synthetic cathinones (e.g., mephedrone, metaphedrone/3-Methylmethcathinone) as the most frequently reported substances [1–3]. According to a systematic review and meta-analysis with the objective of assessing the global prevalence of chemsex among GBM, it was identified that the pooled prevalence was 0.22 (95% CI:0.19–0.25) [4].

GBM engage in chemsex for different reasons, including to increase libido, sexual arousal, confidence, disinhibition, and stamina, thereby facilitating longer sexual sessions and sex with multiple partners [1, 5, 6]. Additionally, drugs are used to enhance valued qualities of sexual experiences, such as intensifying physical sensations, increasing perceived partner attractiveness, fostering feelings of intimacy or connection, and enabling sexual exploration that men may feel inhibited from pursuing when sober [1, 5, 6]. However, despite these motivations, chemsex drugs may represent significant risks, mainly when combined or at high doses [7]. The health risks associated with the use of these substances are not universal among chemsex participants [2, 8–10]. The individual risk is associated with different factors, including dosing practices, frequency, tolerance levels, and underlying health conditions [11]. Their use may involve a range of potential harms from minor transient effects to non-fatal intoxications requiring medical attention to severe outcomes. For instance, synthetic cathinones are associated with acute psychiatric, neurological, and cardiovascular complications, including agitation, paranoia, hallucinations, anxiety, suicidal ideation, tachycardia, hypertension, hyperthermia, and, in severe cases, excited delirium, multiorgan failure, and death [11]. Methamphetamine use is associated with high risk of dependence, cardiovascular toxicity (e.g., cardiomyopathy, arrhythmias, acute coronary syndromes), psychiatric disorders (including depression, anxiety, and psychosis), and long-term neurocognitive impairments affecting memory and executive function [11]. GHB/GBL carries a narrow margin between desired and toxic doses, with risks of overdose leading to profound sedation, hypothermia, coma, and death, as well as the development of dependence and persistent cognitive and emotional regulation deficits following repeated intoxications [11–14].

**Fig. 1:**
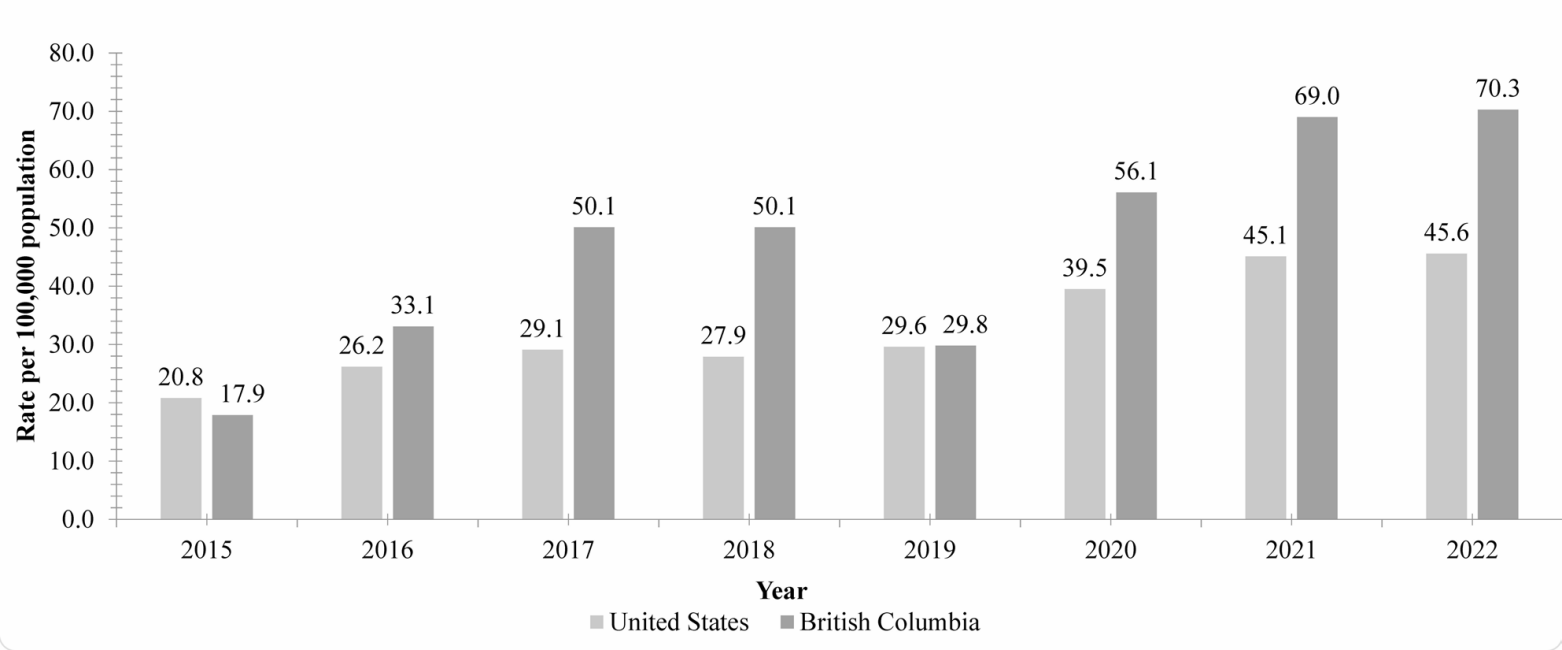
Total overdose deaths among men in the United States and British Columbia (Canada) from 2015 to 2022 [16, 17]. The United States rates reflect all drug overdose deaths. The British Columbia rates reflect all unregulated drug deaths.

Despite a recent growing body of literature studying this phenomenon and its association with sexual risk behaviors, sexually transmitted infections, HIV, and mental health conditions [9], the specific risks associated with overdose remain poorly explored. In Canada, there is an increased concern about the use of illicit substances, with high rates of emergency calls, hospitalizations, and deaths due to opioids and stimulant drug use [15]. From 2016 to 2023, there were 44,592 apparent opioid-related toxicity deaths and 3,479 apparent stimulant toxicity deaths among the general population [15]. Data from the US Centers for Disease Control and Prevention and the British Columbia Coroners Service show a significant increase in the rates of overdose deaths among men from 2015 to 2022 (Fig. 1) [16, 17].

Studies have shown that GBM have higher rates of illicit substance use compared with non-GBM [18–20]. However, it is unclear if the population that most engages in chemsex (i.e., GBM) [21] is also the most affected by these adverse outcomes.

In addition, the recent rise in fentanyl-contaminated stimulants in North America has introduced a significant new risk for chemsex participants, increasing overdose risk [22, 23]. Health Canada’s Drug Analysis Service identified that fentanyl or its analogues have been detected in up to 3% of stimulant-containing drug samples; in British Columbia, this figure rises to 10% [22]. The presence of fentanyl contamination in stimulants may increase the risk of overdose [23]. This contamination can make chemsex practices more hazardous, as participants may unknowingly consume fentanyl and, therefore, be less likely to recognize and effectively counter its effects, such as by using naloxone. It can also lead to cumulative fentanyl exposure as chemsex participants often engage in repeated dosing during prolonged sexual sessions [24], further exacerbating the overdose risk.

## Epidemiology, risks, and barriers to identification

Intoxications from substances commonly used during chemsex are becoming increasingly prevalent. Data collected in sentinel hospitals in the Euro-DEN Plus network indicated that 12.6% of all drug intoxication cases involved GHB/GBL [25]. Among the 12.6% of intoxication cases involving GHB/GBL, 8.4% of individuals who ingested only GHB/GBL required intensive care admission, while those who combined GHB/GBL with other substances accounted for 16.8% of cases [25]. Methamphetamine-associated overdose deaths are also on the rise. From 2013 to 2019 in the US, overdose deaths involving psychostimulants other than cocaine (primarily methamphetamine) increased by 180% among adults aged 18 to 64 years [26]. The use of methamphetamine is significantly higher among GBM compared with other populations [26]. Synthetic cathinones also pose significant risks, particularly when combined with other chemsex-related substances; however, evidence suggests that their overall harm profile is ranked lower compared to methamphetamine and GHB/GBL [27, 28].

Multiple barriers complicate accurately assessing the impact of chemsex on overdose deaths among GBM. First, a lack of systematic documentation of sexual practices in clinical records [29] prevents the identification of chemsex and illicit drug overdose among GBM. Additionally, posthumous identification of GBM who have died from overdose is challenging, as sexual orientation is rarely recorded in medical or legal forms. Second, the chemsex practices that preceded a particular overdose death are rarely known. Stigma may prevent men from disclosing these behaviors [30, 31], and healthcare providers or investigators may fail to inquire about them [32]. In cases of fatal overdose during chemsex, identifying the practice is nearly impossible unless explicitly stated by witnesses or indicated by compelling evidence. Forensic toxicology can identify the substances that potentially caused death, but cannot confirm whether or not the individual was engaged in chemsex. Moreover, toxicology exams might be inconclusive for certain substances, especially those new to the market [12], further complicating efforts to establish a clear connection between chemsex and overdose deaths.

Chemsex practices vary in intensity and frequency [33], which obscures their identification and classification as problematic substance use. Some individuals may perceive their relationship with drug use as problematic; others may not, either because they engage less frequently in chemsex or because they do not perceive their use as harmful within their own framework of risk and benefit [2]. In addition, some participants may understand the risks, accepting the possibility of negative health outcomes as a trade-off for sexual enhancement or community connection [24]. For instance, a study conducted in three Canadian cities identified that 82.6─85.7% of GBM who reported amphetamine use in the last six months reported not feeling they needed help reducing their substance use [34]. This subjective nature of assessing substance use challenges traditional drug treatment programs; consequently, substance use disorders related to chemsex may remain unrecognized or unsupported within conventional care settings. Furthermore, specific aspects of chemsex drug use patterns can increase the risk of overdose. For instance, less frequent participants may lack awareness of their tolerance levels, leading to unintentional overdose [35]. Additionally, the common practice of mixing multiple substances exacerbates the risk of adverse outcomes, including overdose [36]. Public health policies and interventions that fail to address these nuanced risks may inadvertently overlook individuals at higher risk.

## Intervention strategies and research gaps

While opioid substitution therapies, such as methadone or buprenorphine, have demonstrated strong efficacy in reducing opioid-related harms [37, 38], similar pharmacologic treatments for stimulant use disorders remain limited. Medications such as mirtazapine, naltrexone, and bupropion have shown generally modest effects in reducing stimulant use [39, 40]. In contrast to pharmacologic approaches, behavioral strategies, such as contingency management (CM), have shown more consistent effectiveness [41, 42]. CM involves providing tangible rewards contingent on stimulant abstinence and has been associated with reductions in methamphetamine use and improvements in HIV-related outcomes among GBM [43, 44]. Despite its demonstrated efficacy, CM remains underutilized [45].

An example of a CM strategy tailored to stimulant use among GBM is the PnP & Me Program, developed by the Health Initiative for Men (HIM), in Vancouver, Canada, in collaboration with Vancouver Coastal Health and other partners [46]. PnP & Me is a harm-reduction program for gay, bi, queer men and gender-diverse people who use drugs, especially in the context of chemsex. The program integrates harm reduction and sexual health support, offering a peer-led model that may hold value as a replicable practice [46]. The group counselling program provides a supportive space where participants can explore their relationship with substances and establish personal goals, including reducing, changing, or stopping the use of substances. Participants receive incentives for attendance at each group session and for achieving additional personal health goals. PnP & Me participants also have access to free safer-sex and harm reduction supplies through multiple HIM programs [47]. Regardless of pharmacologic therapies for stimulant use, successful interventions must integrate harm reduction with sexual health and mental health or establish strong referral pathways. In addition to stimulant-related risks, GHB/GBL also contributes to overdose vulnerability due to its narrow safety-to-harm window and the potential for rapid loss of consciousness, respiratory depression, and severe intoxication, especially when combined with alcohol and other depressants [48, 49]. Incorporating GHB-specific guidance, including safer dosing education, use destigmatization, and avoidance of co-use with depressants, is also an important component of chemsex harm reduction [49]. Integrated harm reduction approaches are essential to mitigate the potential risks of chemsex, including STI and overdose risks [50]. Key interventions include HIV testing, needle exchange, sexual health screenings, psychosocial interventions, and vaccinations [50]. Community-led initiatives should also be prioritized, ensuring GBM involvement in program design and evaluation [50].

## Conclusion

Data on the prevalence of illicit-drug overdose deaths associated with chemsex among GBM remains limited. Published research on drug-use patterns in this group suggests that they may face unique risks [7]. The lack of robust data underscores the urgent need for targeted research. Future studies should employ mixed-methods approaches that combine quantitative data from longitudinal cohort studies linked to administrative health records with qualitative insights from interviews, focus groups, and medical chart reviews. Such research would provide a clearer understanding of the complex relationship between chemsex and overdose deaths among GBM. By generating robust and actionable evidence, these studies can inform the development of tailored harm reduction strategies, ultimately improving health outcomes and helping to prevent overdose deaths in this population.

## Data Availability

The datasets analyzed during the current study are accessible through the repositories of the US Centers for Disease Control and Prevention and the British Columbia Coroners Service, available at [https://nida.nih.gov/research-topics/trends-statistics/overdose-death-rates](https:/nida.nih.gov/research-topics/trends-statistics/overdose-death-rates) and https://www2.gov.bc.ca/gov/content/life-events/death/coroners-service/statistical-reports.

## Abbreviations

PnP: Party and Play
GBM: Gay, bisexual, and other men who have sex with men
US: United States
PrEP: HIV Pre-exposure prophylaxis
STI: Sexually transmitted infections
GHB/GBL: Gamma-hydroxybutyrate/Gamma butyrolactone
PLWH: People living with HIV
ART: Antiretroviral therapy
CM: Contingency management
HIM: Health Initiative for Men
